# Analysis of Web Spam for Non-English Content: Toward More Effective Language-Based Classifiers

**DOI:** 10.1371/journal.pone.0164383

**Published:** 2016-11-17

**Authors:** Mansour Alsaleh, Abdulrahman Alarifi

**Affiliations:** King Abdulaziz City for Science and Technology (KACST), Riyadh, Saudi Arabia; King Saud University, SAUDI ARABIA

## Abstract

Web spammers aim to obtain higher ranks for their web pages by including spam contents that deceive search engines in order to include their pages in search results even when they are not related to the search terms. Search engines continue to develop new web spam detection mechanisms, but spammers also aim to improve their tools to evade detection. In this study, we first explore the effect of the page language on spam detection features and we demonstrate how the best set of detection features varies according to the page language. We also study the performance of Google Penguin, a newly developed anti-web spamming technique for their search engine. Using spam pages in Arabic as a case study, we show that unlike similar English pages, Google anti-spamming techniques are ineffective against a high proportion of Arabic spam pages. We then explore multiple detection features for spam pages to identify an appropriate set of features that yields a high detection accuracy compared with the integrated Google Penguin technique. In order to build and evaluate our classifier, as well as to help researchers to conduct consistent measurement studies, we collected and manually labeled a corpus of Arabic web pages, including both benign and spam pages. Furthermore, we developed a browser plug-in that utilizes our classifier to warn users about spam pages after clicking on a URL and by filtering out search engine results. Using Google Penguin as a benchmark, we provide an illustrative example to show that language-based web spam classifiers are more effective for capturing spam contents.

## 1 Introduction

Web spamming (or spamdexing) is a process for illegitimately increasing the search rank of a web page with the aim of attracting more users to visit the target page by injecting synthetic content into the page [1, 2]. Web spamming can degrade the accuracy of search engines greatly if this content is not detected and filtered out from the search results [3–5]. In general, spammers aim to illegally enhance the search engine ranks of their spam pages, which might lead to user frustration, information pollution, and distortion of the search results, thereby affecting the entire information search process.

Black hat search engine optimization (SEO) techniques are generally used to create web spam pages. For example, in content-based web spamming, spammers stuff spam keywords into the target page by listing them in the HTML tags (e.g., META tags) or by using an invisible font. In addition, scraper techniques are used where the spam content is simply a replica of another popular site [6–8]. These deception techniques are refused by search engines because they can lead to misleading search results [9].

Some web ranking algorithms give higher ranks to pages that can be reached from other web pages that are highly ranked, so the black hat SEO method exploits this feature to increase the ranks of spam pages [5, 10–13]. For example, in the *cookie stuffing* method, the user’s browser receives a third-party cookie after visiting a spam page with an affiliate site so the cookie stuffer is credited with a commission after visiting the affiliate site and completing a particular qualifying transaction. Moreover, by utilizing a *page cloaking* mechanism, a search engine crawler can receive different content from the spam page compared with that displayed on the end-user’s browser, where the aim is delivering advertisements or malicious content to the user, which is partially or completely irrelevant to that searched for by the user. Another link-based tactic is *link farms* where a set of pages are linked with each other.

Site mirroring is another black hat SEO method, which exploits the fact that many search engines grant higher ranks to pages that contain search keywords in the URL. Thus, spammers can create multiple sites with various URLs but similar content. Further, web spammers can create pages that redirect the user’s browser to a different page that contains the spam content in order to evade detection by search engines [10].

Existing web spam detection approaches use link (e.g., [14]) and content (e.g., [1, 15–18]) information to capture spam pages. For example, Facebook and Twitter filter out messages containing known spam content so they are not posted [19, 20].

Due to the success of email anti-spam tools based on machine learning, we consider that these techniques might also be effective for detecting web spamming. Typically, high detection accuracy and a low false positive rate are the main properties required for detection tools based on machine learning methods. This is particularly important for detecting spam pages and ensuring that benign web sites are not penalized.

Search engines enhance their anti-spamming techniques continuously. For example, Google developed their latest algorithm (called *Penguin*) in 2012 and they have continued updating it to lower the search engine ranks of web sites that use black hat SEO or that violate Google Webmaster Guidelines [21, 22]. Google’s latest web spam report urges publishers to verify the contents of their pages via the Search Console. In fact, Google sent over 4.3 million emails to webmasters during 2015 alone to warn them of identified spam-like content and to give them a chance of reconsideration [23].

The effectiveness of the Google Penguin algorithm is affected by the text language used in the page examined [24]. Several web spam detection features have been proposed but to the best of our knowledge, the effect of the language on these detection features has not been examined previously. In addition, to the best of our knowledge, the performance of the Google Penguin algorithm at detecting web spam pages that contain text in languages other than English has not been evaluated.

This study significantly extends our earlier conference paper [25, 26], where the data set is expanded and updated, a new release of Google Penguin is explored, new spamming detection algorithms are introduced, and their results are presented. This study makes the following main contributions.

1. Effects of language on the detection of web spam. We conducted several experiments to study how the page language affects the detection accuracy and false positive rate, as well as showing how and why the distribution of selected detection features differ according to a given page language. We used English and Arabic as languages in case studies.
2. Collecting an Arabic web spam data set. We collected and manually labeled a corpus containing both benign and spam pages with Arabic content. We used this corpus to evaluate our proposed machine learning-based classifier and we have also made the corpus available for use by the research community in this domain.
3. Analysis of detection features and development of a novel classifier. Using Arabic pages in a case study, we showed how to identify a set of web spam detection features with satisfactory detection accuracy. Employing supervised machine learning techniques, we then built a classifier for detecting web pages that contain spam content and showed that it yielded better accuracy compared with the Google Penguin algorithm.
4. Construction of a browser anti-web spam plug-in. Using our proposed classifier, we developed a browser plug-in to warn the user before accessing web spam pages (i.e., after clicking on a link from the search results). The plug-in is also capable of filtering out spam pages from the search engine results.

The remainder of this paper is organized as follows. Section 2 presents our analysis of how the page language affects the detection rate for web spam using a set of classifiers. Section 3 describes the collection and labeling process for our data set. Section 4 illustrates our system architecture and design. Section 5 explains the feature extraction and selection process. Section 6 presents the proposed classifier and evaluations of its accuracy. Section 7 discusses the meaning and implications of our main findings, and Section 8 presents related research. Finally, we give our conclusions in Section 9.

## 2 Effects of the Page Language

### 2.1 Data Sets

Two web spam data sets were used in this study. First, we used UK-2011 [27], which is a subset of the WEBSPAM-UK2007 data set [28]. The UK-2011 data set was labeled by volunteers and each page is flagged as either “spam” or “non-spam.” Second, we used an extended Arabic web spam data set [29], which included spam and non-spam Arabic pages (this data set was collected and labeled during the period from April 2011 to August 2011).

We used Wahsheh’s web spam detection features [30] (see Table 1). We employed the J48 classifier, which is a Weka (version 3.7.6) implementation of the C4.5 decision tree classifier (decision trees are statistical machine learning algorithms that utilize a greedy top-down process to select attributes at selected nodes in the tree and divide the samples into subsets based on the values of these attributes). Cross-validation, a model evaluation method used to improve how a classifier generalizes to an independent data set, was used to ensure that each instance in the data set had an equal probability of appearing in either the training or testing sets. We performed a 10-fold cross-validation and we divided the data set into 10 chunks for training 10 times, where a different chunk was used as the testing set each time. For the decision tree classifier, the issue of overfitting was addressed by using a *pruning technique*, where the less significant tree nodes for classifying the data set instances were removed from the tree (we set the minimum number of instances to two).

**Table 1:** Feature descriptions used in our study for the effects of the page language on the spam detection rate. Note that the numbers are per page.

Feature No.	Feature Description
1	existing amount of visible and clickable text in a hyperlink (i.e., anchor text)
2	number of words
3	average word length
4	number of words in the *title* elements (because spammers tend to use unrelated characters to enhance the page rank
5	page compression rate
6	number of unique words
7	number of characters in the *meta*-element (because spammers tend to utilize keyword stuffing to enhance the page rank)
8	number of words in the *meta* element (because spammers tend to utilize keyword stuffing to enhance the page rank)
9	longest word (because spammers tend to utilize long words to increase the page rank)
10	shortest word (because spammers tend to utilize long words to increase the page rank)
11	number of images

### 2.2 Results and Analysis

**Fig 1:**
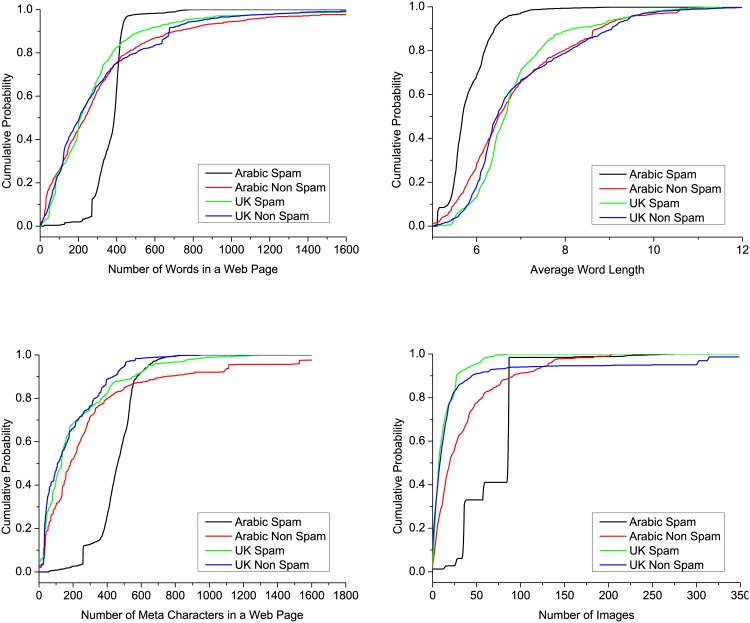
Cumulative distribution function (CDF) for different features in both data sets.

We started our analysis by studying the selected detection features in both data sets. Fig 1 shows the probability density function (PDF) for different features in both data sets. A random sample of 1,500 web pages was used to determine the figure visibility (compared with 3,688 pages in data set (1) and 9,988 in data set (2)). According to the cumulative distribution function (CDF) for feature 2 in Fig 1A, almost 60% of the Arabic non-spam pages contained less than 270 words in their pages, whereas less than 15% of Arabic spam pages had less than 270 words. The figure shows that Arabic spam pages tended to have more words in their pages compared with Arabic non-spam pages. In addition, the CDFs for the number of words in Arabic non-spam pages and English pages were very similar. The same observation can be made based on Fig 1B and 1C, but there was more variation among them. In fact, most of the features exhibited greater variation between spam and non-spam pages in the Arabic data set compared with the UK data set. Furthermore, Fig 1B shows that Arabic spam pages tended to have shorter word lengths, where almost 80% of the Arabic spam pages had an average word length of six characters, whereas only 40% of the Arabic non-spam pages had an average word length of six characters. In terms of the number of characters per meta-element, as shown in Fig 1C, Arabic spam pages usually had more characters (80% had more than 400 characters) compared with Arabic non-spam pages (20% had more than 400 characters). Furthermore, Fig 1D shows that Arabic pages usually had more images in their pages compared with English pages, particularly in spam pages.

First, we used all 11 detection features to build the classifiers. Most of the Arabic web spam pages used more obvious spamming tactics compared with those in English, so the DR for English spam pages was lower than that for those in Arabic. We then selected different sets of features using the following feature selection algorithms implemented in Weka: CfsSubsetEval, PrincipalComponents, ConsistencySubsetEval, and FilteredSubsetEval. Brief descriptions of these algorithms and the results obtained from their execution are shown in Table 2. The CfsSubsetEval algorithm considers the individual predictive ability of every feature as well as the features’ degree of redundancy in order to evaluate the value of a subset of features. PrincipalComponents performs principal components analysis and transforms the data. Based on the results obtained by these algorithms, we selected the following sets as training scenarios for the classifier: 1,5,7,11, 1,5,8,9, 1,5,7,10,11, and all 11 features.

**Table 2:** Results obtained after applying the feature selection algorithms to both data sets.

Attribute evaluator	Search method	Selected features	
		Arabic data set	English data set
CfsSubsetEval	GreedyStepwise	1,5,7,10,11	1,2,3,5,6,7,8,11
PrincipalComponents	Ranker	1,2,3,4,5,6,7,8,9	1,2,3,4,5,6,7,8
ConsistencySubsetEval	GreedyStepwise	1,5,8,9	1,2,3,5,6,8,9,11
FilteredSubsetEval	GreedyStepwise	1,5,7,11	1,2,3,5,6,7,8,11

Tables 3 and 4 show the performance of each set of features using the classifiers described above, the performance measurement indices mentioned in Table 5, and the confusion matrix obtained by the classifier.

**Table 3:** Performance of the decision tree classifier using different sets of features (where S = spam and NS = non-spam).

Data set	Set of features	DR	ER	TP		FP		Precision		Recall		F-measure	
				S	NS	S	NS	S	NS	S	NS	S	NS
English	1,5,7,11	77.22	22.78	0.79	0.75	0.25	0.21	0.78	0.76	0.79	0.75	0.79	0.76
	1,5,8,9	79.74	20.26	0.81	0.78	0.22	0.19	0.81	0.79	0.81	0.78	0.81	0.78
	1,5,7,10,11	78.41	21.59	0.8	0.77	0.24	0.20	0.79	0.77	0.80	0.77	0.80	0.77
	All 11 features	88.13	11.87	0.89	0.87	0.13	0.11	0.88	0.88	0.89	0.87	0.89	0.87
Arabic	1,5,7,11	99.52	0.48	1	0.99	0.01	0	0.99	1	1	0.99	1	1
	1,5,8,9	99.23	0.77	0.99	0.99	0.01	0.01	0.99	0.99	0.99	0.99	0.99	0.99
	1,5,7,10,11	99.52	0.48	1	0.99	0.01	0	0.99	1	1	0.99	1	1
	All 11 features	99.21	0.79	0.99	0.99	0.01	0.01	0.99	0.99	0.99	0.99	0.99	0.99

**Table 4:** Confusion matrix obtained by the decision tree classifier using different sets of features (where S = spam, NS = non-spam).

Data set	Set of features	S		NS	
		S	NS	S	NS
English	1,5,7,11	79.03	20.97	24.83	75.17
	1,5,8,9	81.43	18.57	22.17	77.83
	1,5,7,10,11	80.08	19.92	23.47	76.53
	All 11 features	89.34	10.66	13.24	86.76
Arabic	1,5,7,11	99.7	0.3	0.66	99.34
	1,5,8,9	99.28	00.72	0.82	99.18
	1,5,7,10,11	99.7	0.3	0.66	99.34
	All 11 features	99.44	0.56	1.02	98.98

**Table 5:** Performance measurement indices.

Measurement Indices	Description
Detection rate (DR)	Ratio of the number of correctly classified samples relative to the total number of samples.
Error rate (ER)	Ratio of the number of incorrectly classified samples relative to the total number of samples.
True positive (TP) for class *x*	Ratio of the number of correctly classified samples in class *x* relative to the total number of samples.
False positive (FP) for class *x*	Ratio of the number of incorrectly classified samples in class *x* relative to the total number of samples.
Precision for class *x*	Ratio of the number of correctly flagged samples in class *x* relative to the total number of samples in class *x*.
Recall for class *x*	Ratio of the number of correctly flagged samples in class *x* relative to the total number of correctly classified samples.
F-measure for class *x*	The harmonic mean of precision and recall for class *x*.

### 2.3 Limitations in Existing Data Sets

We found that the distributions of a selected set of features varied according to the underlying language used in the page examined. In addition, for both data sets, the results obtained by the classifiers showed that only a few common features yielded similar results. However, the significance of several of the remaining features varied according to the language used in the page examined. The effect of language was due partly to the use of a similar set of web spamming techniques for a given language.

It is important to note that these data sets are fairly old and they do not represent the current techniques of new spammers. In addition, given that the original contents of the web pages of the two data sets were not available, we could not examine other spam detection features (i.e., other than those of the 11 features provided within the two data sets). Furthermore, the method used to collect the web pages in these data sets did not consider specific search engines as the main goal of spammers in order to obtain higher ranks for their web pages in the search engine results and increase the number of hits. To overcome these limitations, we decided that a new data set must be collected carefully and made available.

## 3 Building an Arabic web spam corpus

**Fig 2:**
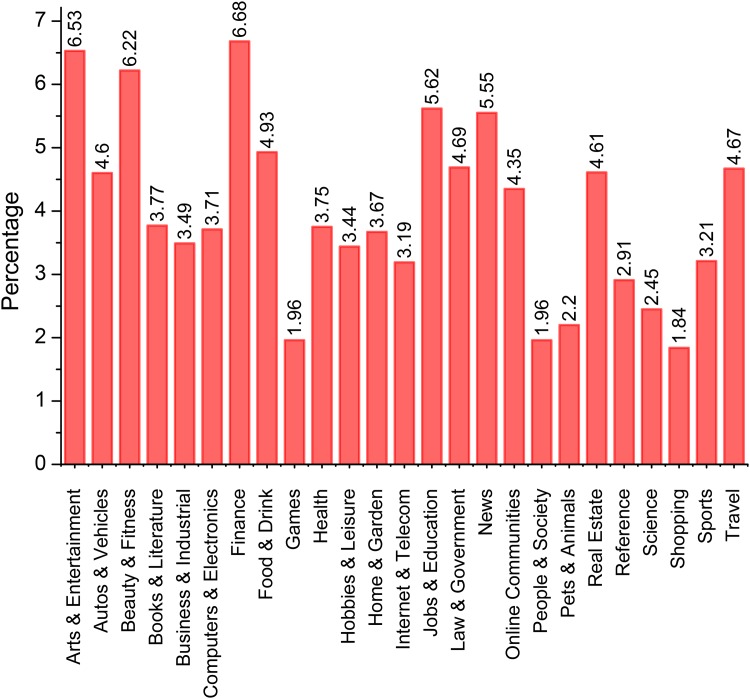
Percentages of the collected URLs in each Google Trends category.

In order to overcome the limitations described in the previous section, we followed a three-step process to collect a data set of Arabic pages, including both benign and spam web pages. First, we collected the top Arabic search keywords for the period from January 2004 to October 2012 on the Google Trends website. We then queried the Google search engine using the collected search keywords. The URLs of the top 50 result pages for each search keyword were then stored, thereby obtaining a total of 8,168 distinct domain names. Fig 2 shows the percentages of the URLs collected for each category in Google Trends. We note that the number of search keywords in a given category affected the corresponding percentage.

**Fig 3:**
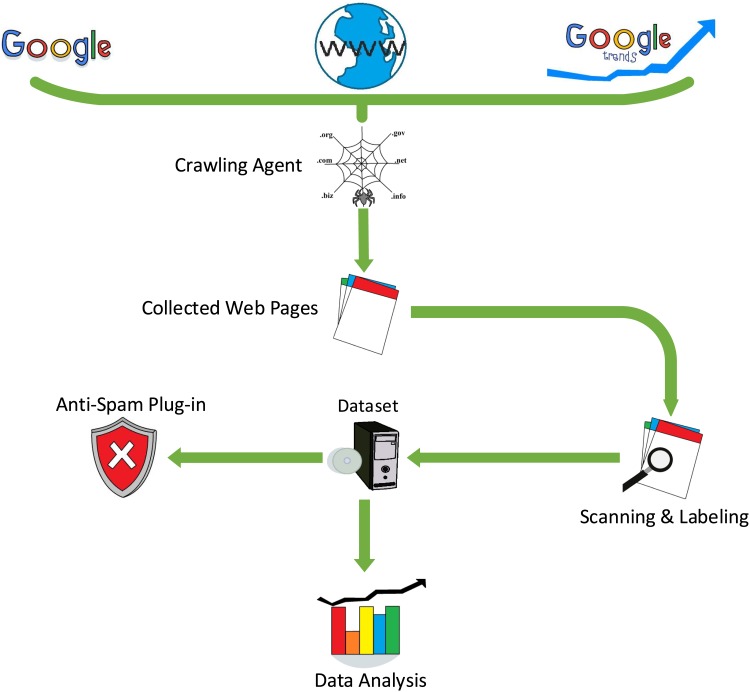
Process flow employed for collecting and building our web spam corpus.

We identified multiple types of pages with malware and phishing content, where each URL was examined using six security scanners (these scanners were provided by selected antivirus vendors): 1) Sucuri SiteCheck scanner; 2) McAfee SiteAdvisor scanner; 3) Google Safe Browsing scanner; 4) Norton scanner; and 5) Sophos scanner (with Yandex ranking). The scanners examined every visible web page in the entire domain of a given URL. This scanning process was beneficial for studying the relationships between existing vulnerabilities, malicious content, and web spam [31]. The scanning results were then stored into a database (see Fig 3).

**Fig 4:**
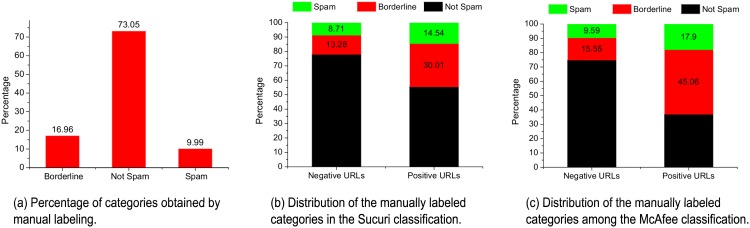
Distribution of the URL categories in the data set.

Finally, the URLs were labeled manually by several raters. Each link was classified into one of four categories: i) spam class; ii) borderline class; iii) benign class; and iv) unknown class. The raters were given a set of guidelines for labeling web spam pages (e.g., see [32]). A web application was utilized by the raters to view and rate the data set’s links so every link was classified by *at least* one rater. Fig 4A shows the distribution of classes (i.e., non-spam, borderline, and spam) according to the raters. It should be noted that almost 26% of the Google search results were flagged as either the spam class (10%) or borderline class (16%), although the new update to the Penguin algorithm has been in place for several months.

Many spammers aim to compromise the machines of users and there was a clear correlation between spamming and the existence of web vulnerabilities, as shown in Fig 4B and 4C. We note that 15% of the positive URLs results obtained from the Sucuri scanner (i.e., containing malware and flagged as malicious) were manually labeled as spam, whereas 9% of the negative web pages were labeled as spam. Similarly, the percentage of URLs flagged as borderline represented (1) 13% of the Sucuri scanner-negative URLs and (2) 30% of the Sucuri scanner-positive URLs. However, the percentage of non-spam URLs represented more than 78% of the negative URLs and 55% of the positive URLs. Similar observations can be made for the sites scanned by the McAfee tool, as shown in Fig 4C, which indicates that spamming seems to be a preferred tool for attackers.

**Fig 5:**
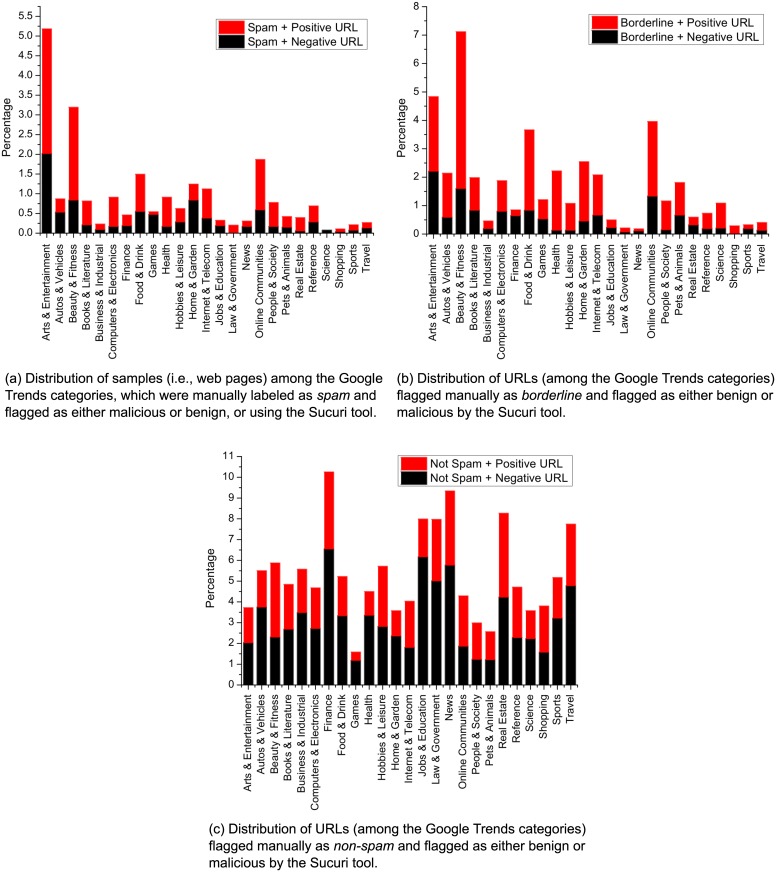
Distribution of positive and negative URLs for different manually labeled categories.

Fig 5A, 5B and 5C illustrate the distributions of our three classes among Google Trends categories. The distribution is divided into two sets: malicious and benign, as found in the URL classification by the Sucuri scanner. The *arts & entertainment*, *beauty & fitness*, and *online communities* categories were most common for web spammers. Furthermore, we note that the numbers of positive and negative URLs according to the Sucuri scanner were proportional to those in the spam and borderline classes, unlike the non-spam category class.

## 4 System Architecture and Design

**Fig 6:**
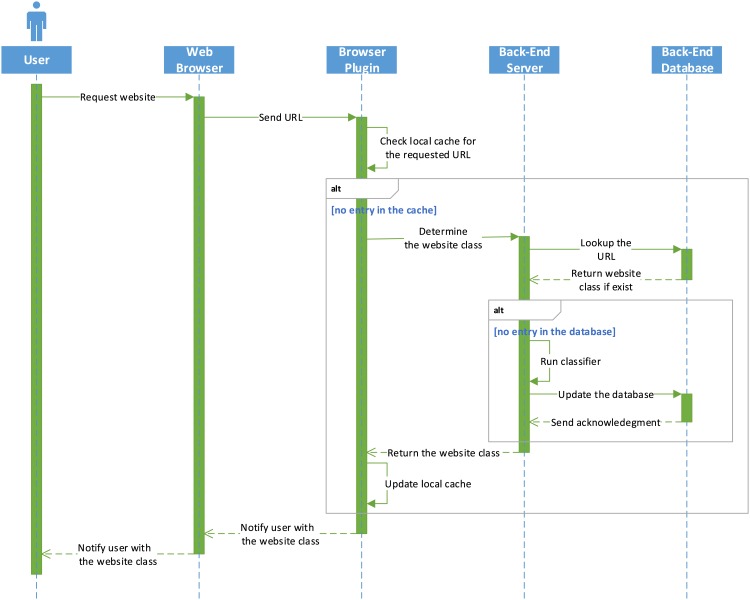
System sequence diagram.

The system comprises two major components: (i) a back-end server and (ii) a browser plug-in. The plug-in represents the connection between the back-end server and the browser (see Fig 6). After the browser plug-in captures the URL (either clicked on or entered in the web browser address bar by the user), the URL is sent by the plug-in to the back-end server, which then extracts the values of the detection features from the URL and flags it as either benign or spam.

The page will be blocked by the plug-in if it is flagged as a spam page and it will display a pop-up dialog box to warn the user of spam content. The user has the option to proceed and browse the spam page. The plug-in maintains a cache with a blacklist and whitelist, so only new URLs are examined by the back-end server. A database containing all the requests received from the plug-ins is also maintained by the back-end server, which serves as a local cache lookup mechanism to speed up the retrieval process.

The plug-in was implemented for the Chrome browser using standard web techniques, such as HTML, CSS, and JavaScript, and JavaScript Object Notation (JSON) is used for lightweight data interchange with the browser and the back-end server. The back-end server uses Apache tomcat as a web server, MySQL as a database server, and JavaServer Pages (JSP) as a server-side programming technology. In the back-end server, jsoup is used as a Java library to deal with HTML and xml document parsing and feature extraction. Most computations are performed on the server side, which maintains a cache containing both the blacklist and whitelist, so the waiting time tends to be very short compared with the loading time for the pages examined. Furthermore, the back-end server can easily be scaled up or down to serve the number of requests. The back-end server can also be used to collect crash reports from the plug-in, which may help to improve new releases.

## 5 Feature Selection and Extraction

Feature selection and extraction are crucial steps in the construction of a classifier. Several previous studies have proposed the detection of features that minimize the intra-class variability and maximize the inter-class variability (e.g., [33–38]). In general, the use of raw data for classification leads to classifiers with complex structures, thereby resulting in poor performance.

**Fig 7:**
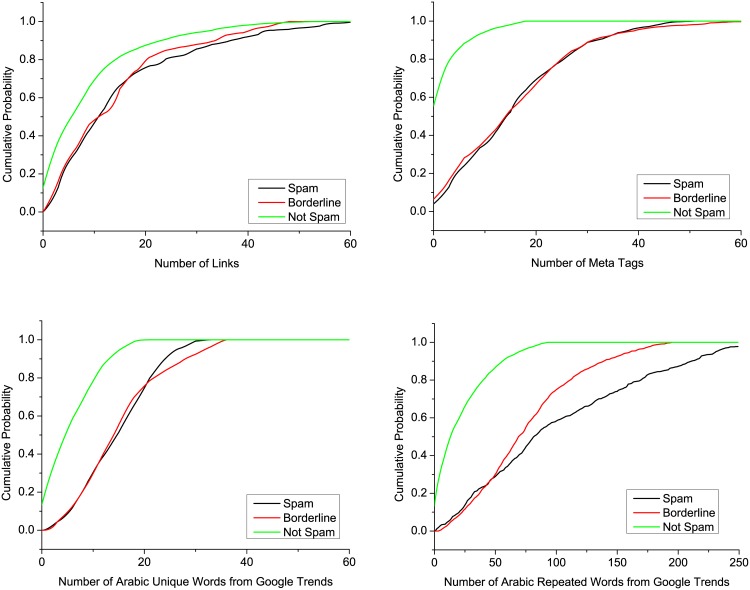
Cumulative distribution function (CDF) for features 2, 5, 6, and 7 in the spam, borderline, and non-spam categories.

In addition to some known features from previous studies, we propose novel detection features that have not been used before to the best of our knowledge, as shown in Table 6. We calculated the CDF for the second feature in Fig 7A, the fifth feature in Fig 7B, the sixth feature in Fig 7C, and feature 7 in Fig 7D, thereby helping us to understand the nature of each feature, and thus the contribution of the features to the classifier’s accuracy.

**Table 6:** Descriptions of the detection features.

Feature No.	Feature Name	Description	Return value	Time comp.
1	Hidden iframes	Number of inline frames used to embed other documents within the current HTML document	Integer	*O*(*n*)
2	Number links	Number of hyperlinks	Integer	*O*(*n*)
3	Doorway pages	Indicates whether the page redirects visitors without their knowledge	Boolean	*O*(*n*)
4	Meta refresh	Indicates whether the page contains meta-refresh, which is used to automatically refresh the page after a given time interval and redirect it	Boolean	*O*(*n*)
5	Meta tag	Number of meta tags comprising part of the page’s header and that provide metadata about the page	Integer	*O*(*n*)
6	Word_Stuffing_n	Number of unique words from Google Trends in the page	Integer	*O*(*n*^2^)
7	Word_Stuffing_r	Number of repeated words from Google Trends in the page	Integer	*O*(*n*^2^)

As shown in Fig 7A, 70% of the web spam and borderline pages had ≤18 links, whereas the benign pages had ≤ 10 links. Fig 7B shows that 90% of the benign web pages had ≤ 8 meta tags compared with ≤ 37 meta tags in the borderline and spam pages.

**Fig 8:**
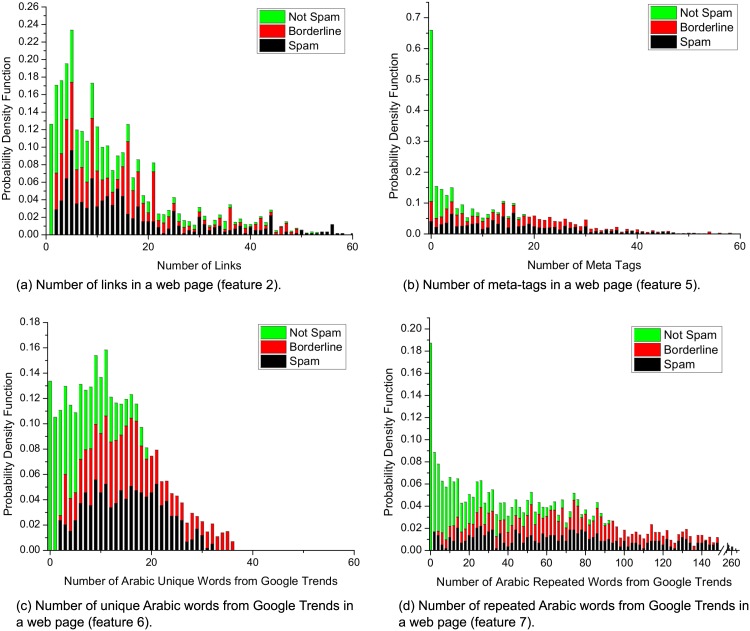
Probability density function (PDF) for features 2, 5, 6, and 7 in the spam, borderline, and non-spam categories.

Similarly, Fig 7C and 7D show clearly that for features 6 and 7, the benign web pages were sufficiently easy to distinguish from both borderline and spam web pages. For instance, 90% of the benign pages had 12 unique words from Google Trends compared with 25–30 words in both the borderline and spam pages. Furthermore, 90% of the benign web pages had 70 repeated words from Google Trends compared with 170–230 words in both the borderline and spam web pages. Features 6 and 7 were actually critical for distinguishing between spam and borderline URLs. In almost 50% of cases, the borderline and spam web pages differed from each other by 50–60 words (see Fig 7D). We also calculated the PDF for the same features, as shown in Fig 8.

**Fig 9:**
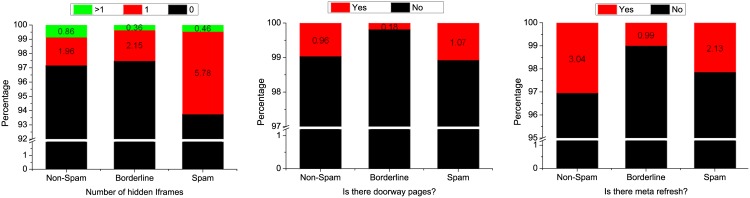
Distributions of features 1, 3, and 4 in the spam, borderline, and non-spam categories.

Fig 9A shows that 6% of the spam pages had one hidden iframe, whereas this was the case for only 2% of the borderline and benign pages. It should be noted that although some detection features might not prove useful in isolation, employing multiple features for detection could result in better detection performance when distinguishing between benign and spam pages because these features may complement each other (see Fig 9B and 9C).

**Fig 10:**
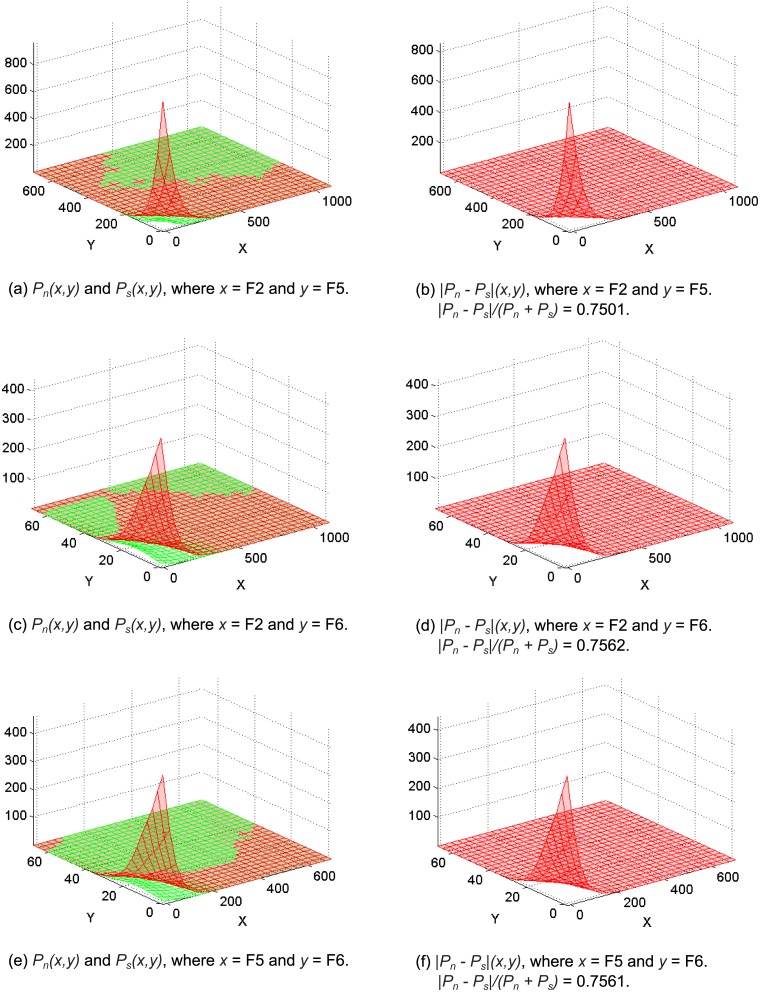
Probability density functions *P*_*n*_ and *P*_*s*_ for different combinations of features, where *n* denotes non-spam pages (in red) and *s* denotes spam pages (in green).

Fig 10 shows the PDFs for some selected combinations of detection features where the difference between non-spam and spam pages was significant. In Fig 10A, we note that there is one obvious peak where the PDF for the non-spam pages was much greater than that for the spam pages (the x-axis represents feature *F*2 and the y-axis represents feature *F*5, as in Table 6; the non-spam class is shown in red and the spam class in green). Fig 10B shows the delta values (i.e., |*P*_*n*_ − *P*_*s*_|(*F*2, *F*5)). Similarly, in Fig 10C, when the values of features *F*2 and *F*6 were relatively small, there was a clear peak where the PDF for the non-spam pages was greater than that for the spam pages. Fig 10D shows the delta values (i.e., |*P*_*n*_ − *P*_*s*_|(*F*2, *F*6)). Similar observations can be made based on Fig 10E and 10F.

## 6 Classification and Evaluation

We tested four machine learning algorithms by using multiple variations to build our classifier, as follows. First, we tested decision trees (C4.5, logistic model tree, random forest, and LogitBoost). Second, we tested Bayes Network, which is a probabilistic graphical model that represents the relationships and conditional dependencies between a set of random variables using a graphical model. Third, we tested a support vector machine (SVM), a statistical-based algorithm that separates classification classes using a set of hyperplanes. Fourth, we tested a multilayer neural network, which comprises a set of interconnected processing units (the weights of these interconnections are calibrated during the training phase to obtain the required knowledge).

Understanding the similarity between spam and borderline web pages is important for the prior training of classification models (see Section 5). To build our classifiers, we considered the following scenarios: (i) two-class classification with only two classes: class 1 for spam and borderline web pages, and class 2 for benign pages; and (ii) three-class classification where we had three classes: spam pages, borderline pages, and benign pages.

The classifiers were configured using Weka (version 3.7.6) for both scenarios [39]. The parameters settings for the three algorithms are shown in Table 7. We performed 10-fold cross-validations for each of the classifiers by using a subset of the observations to establish the classifier and to identify whether the classifier correctly flagged the eliminated observations. To address the overfitting problem for the decision tree classifier, we utilized a *pruning technique* to reduce the size of the tree by eliminating tree nodes with low significance for classifying instances. Pruning techniques are used for reducing the complexity of classifiers, which in turn helps to reduce the time required to execute the classifier in the browser plug-in. For the other classifiers, a validation threshold was used to stop the training process when the algorithm detected overfitting and misclassification increased in the validation set. In order to deal with an imbalanced data set, we used the Synthetic Minority Oversampling Technique (SMOTE), which is an oversampling technique for the minority in an imbalanced data set based on the use of “synthetic” examples. The letter “S” is used at the end of the abbreviations in the tables to indicate whether SMOTE was applied to the data set or not.

**Table 7:** Parameters used in the decision tree, Bayesian network, support vector machine (SVM), and multilayer neural network methods (see Part II of the WEKA Manual for descriptions of the various algorithms used in our study [40]).

Parameter	Classification Model								
	Decision Tree				Bayesian Network	SVM		Multilayer Neural Network	
Training Tool	Weka 3.7.6								
Algorithm	J48 (C4.5)	Logistic Model Tree	Random Forest	LogitBoost	Bayesian Network	SMO (SVM)		Multilayer perceptron trained with gradient descent method (MLP-GD)	
Abbreviation	J48	LMT	RFT	LBT	BayesNet	SMO-P	SMO-R	MLP-GD10	MLP-GD20
Classifier				Decision stump	Simple estimator				
Search Algorithm					Simulated annealing			Gradient descent	
Confidence Factor	0.25								
Number of trees			10						
Maximum depth			∞						
Minimum number of instances per leaf	2								
Pruning	Yes								
Validation technique	Cross-validation with 10 folds								
Training time								500 epochs	
Learning rate								0.3	
Momentum								0.2	
Data normalization						Yes		Yes	
Number of hidden layers								1	
Neurons in the hidden layer								10	20
Activation function								sigmoid	
Validation threshold								20	
Complexity parameter (c)						1			
Kernel function						Poly.	RBF		
Balancing approach	Synthetic Minority Oversampling TEchnique (SMOTE)								
others	kept as default								

The results obtained after training the classifier in the three-class scenario are shown in Table 8, which demonstrate that decision trees performed the best, followed by the Bayesian network, multilayer neural network, and SVM classifiers. In particular, the random forest (RFT-S) decision tree scores were better than those produce by all of the other algorithms, with the highest precision (value of 84%), F-measure (value of 84%), and ROC (value of 95%) scores. The LMT-S decision tree scores were second best with precision and F-measure values of 79%, and ROC = 89%.

**Table 8:** Classification accuracy for three classes.

Algorithm	Performance Measurement Indices—Three Classes																			
	DR	ER	TP			FP			Precision			Recall			F-measure			ROC		
			Spam	Borderline	Non-spam	Spam	Borderline	Non-spam	Spam	Borderline	Non-spam	Spam	Borderline	Non-spam	Spam	Borderline	Non-spam	Spam	Borderline	Non-spam
**J48**	78.02	21.98	0.35	0.42	0.93	0.04	0.07	0.44	0.49	0.55	0.85	0.35	0.42	0.93	0.40	0.48	0.89	0.74	0.72	0.82
			0.78			0.33			0.76			0.78			0.77			0.79		
**J48-S**	78.33	21.67	0.79	0.72	0.85	0.11	0.12	0.1	0.78	0.76	0.81	0.79	0.72	0.85	0.78	0.74	0.83	0.87	0.84	0.91
			0.78			0.11			0.78			0.78			0.78			0.87		
**LMT**	78.63	21.37	0.29	0.43	0.94	0.03	0.07	0.46	0.52	0.56	0.84	0.29	0.43	0.94	0.38	0.48	0.89	0.81	0.80	0.86
			0.79			0.35			0.76			0.79			0.77			0.85		
**LMT-S**	78.83	21.17	0.78	0.73	0.85	0.10	0.12	0.10	0.79	0.76	0.81	0.78	0.73	0.85	0.79	0.75	0.83	0.89	0.86	0.92
			0.79			0.11			0.79			0.79			0.79			0.89		
**RFT**	78.36	21.64	0.43	0.49	0.91	0.05	0.09	0.37	0.52	0.54	0.87	0.43	0.49	0.91	0.47	0.51	0.89	0.83	0.80	0.87
			0.78			0.28			0.77			0.78			0.78			0.85		
**RFT-S**	83.96	16.04	0.87	0.80	0.85	0.09	0.08	0.07	0.84	0.83	0.86	0.87	0.80	0.85	0.85	0.81	0.86	0.96	0.93	0.95
			0.84			0.08			0.84			0.84			0.84			0.95		
**LBT**	78.28	21.72	0.27	0.47	0.93	0.03	0.09	0.43	0.52	0.53	0.85	0.27	0.47	0.93	0.35	0.50	0.89	0.83	0.82	0.87
			0.78			0.33			0.76			0.78			0.77			0.86		
**LBT-S**	62.51	37.49	0.49	0.63	0.75	0.15	0.29	0.12	0.62	0.52	0.75	0.49	0.63	0.75	0.55	0.57	0.75	0.79	0.74	0.89
			0.63			0.19			0.63			0.63			0.62			0.81		
**BayesNet**	78.39	21.61	0.24	0.46	0.94	0.02	0.08	0.46	0.54	0.54	0.84	0.24	0.46	0.94	0.33	0.50	0.89	0.83	0.83	0.87
			0.78			0.35			0.76			0.78			0.76			0.86		
**BayesNet-S**	74.32	25.68	0.71	0.59	0.93	0.15	0.12	0.12	0.70	0.71	0.8	0.71	0.59	0.93	0.71	0.65	0.86	0.88	0.84	0.96
			0.74			0.13			0.74			0.74			0.74			0.89		
**SMO-P**	75.36	24.64	0.01	0.23	0.99	0	0.03	0.80	0.5	0.63	0.76	0.01	0.23	0.99	0.01	0.33	0.86	0.50	0.61	0.59
			0.75			0.58			0.71			0.75			0.68			0.59		
**SMO-P-S**	54.53	45.47	0.17	0.66	0.80	0.04	0.37	0.27	0.68	0.47	0.6	0.17	0.66	0.80	0.28	0.55	0.69	0.67	0.65	0.78
			0.55			0.23			0.58			0.55			0.50			0.70		
**MLP-GD10**	77.66	22.34	0.22	0.49	0.93	0.03	0.10	0.41	0.49	0.50	0.85	0.22	0.49	0.93	0.30	0.49	0.89	0.81	0.82	0.87
			0.78			0.32			0.75			0.78			0.76			0.85		
**MLP-GD10-S**	60.15	39.85	0.56	0.49	0.76	0.22	0.22	0.16	0.56	0.53	0.71	0.56	0.49	0.76	0.56	0.51	0.73	0.77	0.73	0.87
			0.60			0.20			0.60			0.60			0.60			0.79		
**MLP-GD20**	77.59	22.41	0.22	0.48	0.93	0.03	0.1	0.42	0.47	0.50	0.85	0.22	0.48	0.93	0.30	0.49	0.89	0.81	0.82	0.86
			0.78			0.32			0.75			0.78			0.76			0.85		
**MLP-GD20-S**	60.74	39.26	0.54	0.52	0.77	0.20	0.24	0.16	0.58	0.52	0.71	0.54	0.52	0.77	0.56	0.52	0.74	0.77	0.73	0.88
			0.61			0.20			0.60			0.61			0.61			0.79		

However, we note that the detection accuracy was relatively low due partly to two main causes: (1) the URLs in the spam and borderline classes (27% of the data set) were actually similar; and (2) the fact that spammers use clever tactics to evade detection by Google Penguin. For mitigation purposes, we only established the classification models for the two-class scenario. Table 9 shows that the performance of decision tree was better than that of the other classifiers (particularly the RFT-S algorithm where DR = 87% and ROC = 93%). Similarly, the BayesNet-S classifier was ranked second, where DR = 86% and ROC = 93%, followed by the multilayer neural network and SVM classifiers.

**Table 9:** Classification accuracy for two classes.

Algorithm	Performance Measurement Indices—Two Classes													
	DR	ER	TP		FP		Precision		Recall		F-measure		ROC	
			Spam	Non-spam	Spam	Non-spam	Spam	Non-spam	Spam	Non-spam	Spam	Non-spam	Spam	Non-spam
**J48**	84.00	16.01	0.61	0.93	0.07	0.39	0.77	0.86	0.61	0.93	0.68	0.89	0.86	0.86
			0.84		0.30		0.84		0.84		0.83		0.86	
**J48-S**	84.30	15.70	0.85	0.84	0.16	0.15	0.84	0.85	0.85	0.84	0.84	0.84	0.89	0.89
			0.84		0.16		0.84		0.84		0.84		0.89	
**LMT**	83.65	16.35	0.61	0.93	0.07	0.40	0.61	0.86	0.61	0.93	0.67	0.89	0.86	0.86
			0.84		0.31		0.83		0.84		0.83		0.86	
**LMT-S**	84.60	15.40	0.85	0.84	0.16	0.15	0.84	0.85	0.85	0.84	0.85	0.85	0.89	0.89
			0.85		0.15		0.85		0.85		0.85		0.89	
**RFT**	83.41	16.59	0.69	0.89	0.11	0.31	0.71	0.88	0.69	0.89	0.70	0.89	0.87	0.87
			0.83		0.26		0.83		0.83		0.83		0.87	
**RFT-S**	87.13	12.87	0.89	0.85	0.15	0.11	0.86	0.89	0.89	0.85	0.87	0.87	0.93	0.93
			0.87		0.13		0.87		0.87		0.87		0.93	
**LBT**	82.57	17.43	0.59	0.92	0.08	0.41	0.73	0.85	0.59	0.92	0.65	0.88	0.87	0.87
			0.83		0.32		0.82		0.83		0.82		0.87	
**LBT-S**	80.61	19.39	0.81	0.80	0.20	0.19	0.81	0.81	0.81	0.80	0.81	0.81	0.87	0.87
			0.81		0.19		0.81		0.81		0.81		0.87	
**BayesNet**	83.35	16.65	0.63	0.91	0.09	0.38	0.74	0.86	0.63	0.91	0.68	0.89	0.87	0.87
			0.83		0.30		0.83		0.83		0.83		0.87	
**BayesNet-S**	86.69	13.31	0.83	0.91	0.09	0.18	0.9	0.84	0.83	0.91	0.86	0.87	0.93	0.93
			0.87		0.13		0.87		0.87		0.87		0.93	
**SMO-P**	80.97	19.03	0.42	0.96	0.04	0.58	0.80	0.81	0.42	0.96	0.55	0.88	0.69	0.69
			0.81		0.43		0.81		0.81		0.79		0.69	
**SMO-P-S**	76.60	23.40	0.71	0.82	0.18	0.29	0.80	0.74	0.71	0.82	0.75	0.78	0.77	0.77
			0.77		0.23		0.77		0.77		0.77		0.77	
**MLP-GD10**	82.39	17.61	0.67	0.88	0.12	0.33	0.69	0.88	0.67	0.88	0.68	0.88	0.86	0.86
			0.82		0.27		0.82		0.82		0.82		0.86	
**MLP-GD10-S**	79.93	20.07	0.77	0.83	0.17	0.23	0.82	0.78	0.77	0.83	0.79	0.81	0.87	0.87
			0.80		0.20		0.80		0.80		0.80		0.87	
**MLP-GD20**	82.65	17.35	0.68	0.88	0.12	0.32	0.69	0.88	0.68	0.88	0.69	0.88	0.86	0.86
			0.83		0.26		0.83		0.83		0.83		0.86	
**MLP-GD20-S**	80.25	19.75	0.77	0.83	0.17	0.23	0.82	0.79	0.77	0.83	0.80	0.81	0.87	0.87
			0.80		0.20		0.80		0.80		0.80		0.87	

Tables 10 and 11 show the confusion matrices (i.e., error matrix) for the three-class and two-class classifiers, respectively. In each confusion matrix, the first row represents the actual class and the second row represents the predicted class or that classified by a given classifier. Thus, for the RFT-S algorithm, the number of correctly detected spam instances (i.e., TPs) was 87, the number of spam instances mistakenly flagged as borderline was seven, and the number of spam instances mistakenly flagged as non-spam was five. Similarly, the number of correctly detected non-spam instances (i.e., true negatives) was 85, the number of non-spam instances mistakenly flagged as borderline was nine, and the number of non-spam instances mistakenly flagged as spam was five.

**Table 10:** Confusion matrix for three-class classifiers.

Classes		Spam			Borderline			Non-spam		
Classified as		Spam	Borderline	Non-spam	Spam	Borderline	Non-spam	Spam	Borderline	Non-spam
**Algorithms**	J48	34.55	24.81	40.64	12.3	42.1	45.6	2.22	4.86	92.92
	J48-S	78.47	14.19	7.34	15.84	71.60	12.56	6.02	9.05	84.93
	LMT	29.22	28.01	42.77	9.96	42.55	47.49	1.39	4.19	94.42
	LMT-S	78.34	14.21	7.45	14.56	73.16	12.28	6.11	8.88	85.01
	RFT	42.92	24.05	33.03	12.57	48.74	38.69	2.72	6.69	90.59
	RFT-S	87.31	7.65	5.04	11.54	79.48	8.98	5.74	9.18	85.08
	LBT	26.64	32.57	40.79	9.43	46.49	44.08	1.24	5.41	93.35
	LBT-S	49.07	41.2	9.73	21.78	63.11	15.11	8.3	16.36	75.34
	BayesNet	24.2	30.75	45.05	8.17	45.51	46.32	1.02	4.89	94.09
	BayesNet-S	70.56	20.12	9.32	26.97	58.97	14.06	2.72	3.84	93.44
	SMO-P	0.46	14.61	84.93	0.18	22.62	77.2	0.02	1.15	98.83
	SMO-P-S	17.38	57.89	24.73	5.09	66.26	28.65	3.13	16.92	79.95
	MLP-GD10	21.46	40.64	37.9	7.99	49.01	43	1.26	6.13	92.61
	MLP-GD10-S	55.93	30.92	13.15	33.34	48.79	17.87	11.56	12.71	75.73
	MLP-GD20	22.07	38.51	39.42	9.16	47.57	43.27	1.28	5.93	92.79
	MLP-GD20-S	53.97	32.64	13.39	30.54	51.57	17.89	8.78	14.55	76.67

**Table 11:** Confusion matrix for two-class classifiers.

Classes		Spam		Non-spam	
Classified as		Spam	Non-spam	Spam	Non-spam
**Algorithms**	J48	60.59	39.41	7.00	93.00
	J48-S	84.77	15.23	16.17	83.93
	LMT	60.47	39.53	7.43	92.57
	LMT-S	85.43	14.57	16.23	83.77
	RFT	68.83	31.17	10.97	89.03
	RFT-S	89.16	10.84	14.91	85.09
	LBT	58.84	41.16	8.30	91.70
	LBT-S	80.82	19.18	19.60	80.40
	BayesNet	62.51	37.49	8.63	91.37
	BayesNet-S	82.54	17.46	91.15	90.85
	SMO-P	42.07	57.93	4.06	95.94
	SMO-P-S	71.09	28.91	17.88	82.12
	MLP-GD10	67.19	32.81	11.76	88.24
	MLP-GD10-S	76.63	23.37	16.78	83.22
	MLP-GD20	68.32	31.68	11.84	88.16
	MLP-GD20-S	77.41	22.59	16.91	83.09

## 7 Further Discussion

In this study, we used two public data sets (see Section 2) to show that spammers who target different languages behave differently and develop their own new tactics to influence the results obtained by search engine ranking algorithms. In fact, this issue has been recognized by search engine companies and they are considering the development of ranking algorithms that are global and language-independent as far as possible in their new releases.

In most web spam data sets, however, search engine ranking algorithms were not considered when the data sets were constructed. In this study, we constructed a new data set to address this issue (see Section 3). Our data set was carefully selected to contain highly ranked Web pages according to the Google Penguin ranking algorithm. However, this data set led to a concern about the effectiveness of the Google anti-spamming algorithm against spam pages containing Arabic content as well as other non-English languages. In particular, when the data set was examined using six security scanners, the results showed that a significant number of these sites contained malicious contents, thereby indicating that Google search only removed some of the reported malicious sites determined by the Web scanners of other anti-virus vendors. Several of these pages were found to also contain Web spam content.

In a further study (see Sections 5 and 6), we explored the effectiveness of multiple detection features using our data set and we evaluated different classifiers. Despite that some of our classifiers obtained a detection rate of 87%, which might be lower than previous reported detection rates in other studies, we demonstrated that spammers employ clever techniques to avoid being detected by Google Penguin. We also confirmed the need to build more representative and realistic data sets that are suitable to the context of the outputs obtained by search engines.

## 8 Related Work

Numerous previous studies have investigated the prevalence of web spam and various detection techniques have been proposed using different approaches. Gyongyi and Garcia-Molina proposed a web spam taxonomy after the web spam problem emerged in the early 2000s [2]. Heymann et al. were the first to survey the detection, demotion, and prevention of web spam [41]. Recent surveys of existing spam detection techniques and mechanisms have analyzed their advantages and disadvantages (e.g., [42] and [43]). It should be noted that spam and automated accounts in social networks have also contributed to the prevalence of web spam (e.g., see [44–48]). The detection features used for web spam in previous studies belong to two categories: (1) those that exploit topology and network-related data; and (2) those that exploit the web page content.

Gyongyi et al. [1] proposed an algorithm for identifying pages that are likely to be spam and those that are likely to be reputable (also see [49] and [50] for improved versions of the algorithm). Fetterly et al. [51] utilized statistical analysis to show that there are outliers in the statistical distribution of the linkage structure, page content, and page evolution properties in spam pages compared with benign web pages. Wu et al. [52] proposed some alternative methods for propagating trust on the web and utilized distrust to demote web spam. In addition, Castillo et al. [53] built a machine learning classifier that utilizes both link-based and content-based detection features, which obtained TP = 88.4% and FP = 6.3%. Svore et al. [33] built a classifier to identify web spam pages by training a SVM classifier based on a selected set of page attributes.

Ntoulas et al. [15] proposed a C4.5 decision tree classifier, which could detect 86.2% of the spam pages examined. Becchetti et al. [37] explored the best combinations of spam detection features and selected classifiers that achieved high precision (DR = 80.4%) using a small set of features. Furthermore, Abernethy et al. [54] proposed a machine learning classifier that employs a variety of SVM for detecting web spam using both the page content and hyperlinks. Similarly, Becchetti et al. [55] proposed a link-based technique for detecting web spam pages by using a damping function for rank propagation and an approximate counting technique. By exploiting textual and extra-textual features in HTML source code, Urvoy et al. [56] investigated multiple HTML style similarity measures and proposed a flexible clustering algorithm for identifying web spam pages. In addition, Gan and Suel [57] proposed a classifier that uses the decision tree C4.5 algorithm and many detection features, including content-based and link-based, which obtained precision of around 88%. Webb et al. [58] identified a relationship between email and web spam, which they utilized to identify web spam. They also employed their method to collect a web spam corpus. Lee et al. [59] proposed a simplified swarm optimization method to solve the complexity problem that affects statistical classification and machine learning approaches, which increases when there are a large number of web spam detection features.

Previous studies also considered linguistic-based detection features and evaluated their effectiveness at web spam classification (e.g., [36, 60]). However, to the best of our knowledge, no previous studies have investigated the advantages of using linguistic-based features to improve web spam detection in a particular language.

## 9 Conclusion and Future Work

Google continues to improve their Penguin algorithm, but web spammers are also developing creative evasion mechanisms to increase their web page ranks with the aim of attracting more users. In fact, we consider that web spam will remain a good method for both phishing attacks and malware spreading. In this study, we showed that Google anti-spamming methods are actually ineffective against web spam pages that contain non-English content, which raises a concern that the insufficient testing of pages with non-English content could potentially encourage spammers to target these pages.

As an illustrative example, we developed and tested a classifier in the form of a browser anti-spam plug-in for detecting Arabic spam pages, and we showed that our classifier captured most of the web spam pages not detected by the Penguin algorithm. We also created a labeled Arabic web spam data set to evaluate our classifier and to encourage other researchers to build upon our work.

In future work, we plan to extend our web spam data set, create similar data sets for other languages, and develop custom classifiers for these languages. Spammers and Google search engine developers are continually improving their techniques to defeat each other, so future experimental studies are important for understanding new trends and directions. In recent years, large-scale spamming campaigns using compromised Web sites have been performed to corrupt search engine results. These spamming campaigns are an emerging trend that needs to be investigated. Using Google Penguin as a benchmark, our illustrative example shows that language-based web spam classifiers are more effective at capturing spam content. We consider that the web spam problem requires a continuous effort from search engines as well as developers and webmasters based on appropriate vetting of their sites, and end-users should also report spam content.

## References

[1] Gyöngyi Z, Garcia-Molina H, Pedersen J. Combating web spam with trustrank. In: Proceedings of the Thirtieth international conference on Very large data bases-Volume 30. VLDB Endowment; 2004. p. 576–587.

[2] Gyongyi Z, Garcia-Molina H. Web spam taxonomy. In: First international workshop on adversarial information retrieval on the web (AIRWeb 2005); 2005.

[3] Castillo C, Davison BD. Adversarial Web Search. Foundations and trends in Information Retrieval. 2011;4(5):377–486. 10.1561/1500000021

[4] Fetterly D. Adversarial Information Retrieval: The Manipulation of Web Content. ACM Computing Reviews. 2007;.

[5] Henzinger MR, Motwani R, Silverstein C. Challenges in web search engines. SIGIR Forum. 2002;36(2):11–22. 10.1145/792550.792553

[6] Thurow S. Search engine visibility. 2nd ed New Riders Publishing; 2008.

[7] Wallace D. Spamming techniques that you will want to avoid;. http://www.searchrank.com/articles/003.html.

[8] Wilkinson T. Just say no to SEO spam;. http://www.w-edge.com/articles/spam.htm.

[9] Malaga RA. Search Engine Optimization—Black and White Hat Approaches. Advances in Computers. 2010;78:1–39.

[10] Wu B, Davison BD. Cloaking and redirection: A preliminary study. In: Proceedings of the First International Workshop on Adversarial Information Retrieval on the Web (AIRWeb); 2005. p. 7–16.

[11] Edelman B. Deterring Online Advertising Fraud Through Optimal Payment in Arrears Springer; 2009 10.2139/ssrn.1095262

[12] Payton AM. A review of spyware campaigns and strategies to combat them. In: Proceedings of the 3rd annual conference on Information security curriculum development. ACM; 2006. p. 136–141.

[13] Edelman B. Ad thumbnails, Advertisers Funding Direct Revenue;. http://www.benedelman.org/spyware/images/dr-mar06/.

[14] Thomas K, Grier C, Ma J, Paxson V, Song D. Design and evaluation of a real-time url spam filtering service. In: Security and Privacy (SP), 2011 IEEE Symposium on. IEEE; 2011. p. 447–462.

[15] Ntoulas A, Najork M, Manasse M, Fetterly D. Detecting spam web pages through content analysis. In: Proceedings of the 15th international conference on World Wide Web. ACM; 2006. p. 83–92.

[16] Benczur AA, Csalogány K, Sarlós T, Uher M. SpamRank–Fully Automatic Link Spam Detection Work in progress. In: Proceedings of the First International Workshop on Adversarial Information Retrieval on the Web; 2005.

[17] Davison BD. Recognizing nepotistic links on the web Artificial Intelligence for Web Search. 2000; p. 23–28.

[18] Gyongyi Z, Berkhin P, Garcia-Molina H, Pedersen J. Link spam detection based on mass estimation. In: Proceedings of the 32nd international conference on Very large data bases. VLDB Endowment; 2006. p. 439–450.

[19] Facebook, “Explaining Facebook’s spam prevention systems”;. http://blog.facebook.com/blog.php?post=403200567130.

[20] F-Secure, “Twitter now ltering malicious URLs”;.

[21] Miller, M. Matt Cutts Talks Google Penguin, Negative SEO, Disavowing Links, Bounce Rate & More; 25 Oct. 2012. http://searchenginewatch.com/article/2182895.

[22] Sullivan D. Two Weeks In, Google Talks Penguin Update, Ways To Recover & Negative SEO; 10 May 2012. http://searchengineland.com/google-talks-penguin-update-recover-negative-seo-120463.

[23] How we fought webspam in 2015.;. https://webmasters.googleblog.com/2016/05/how-we-fought-webspam-in-2015.html.

[24] Cutts M. Another step to reward high-quality sites;. http://insidesearch.blogspot.com/2012/04/another-step-to-reward-high-quality.html.

[25] Alarifi A, Alsaleh M. Web Spam: A Study of the Page Language Effect on the Spam Detection Features. In: the 11th IEEE International Conference on Machine Learning and Applications (ICMLA); 2012.

[26] Alarifi A, Alsaleh M, Al-Salman A, Alswayed A, Alkhaledi A. Google Penguin: Evasion in Non-English Languages and a New Classifier. In: the 12th IEEE International Conference on Machine Learning and Applications (ICMLA); 2013.

[27] UK-2011 Web spam Dataset.;. https://sites.google.com/site/heiderawahsheh/home/web-spam-2011-datasets/uk-2011-web-spam-dataset.

[28] Web Spam UK2007 Dataset.;. http://barcelona.research.yahoo.net/webspam/datasets/uk2007/.

[29] Extended Arabic Web Spam 2011 Dataset.;. https://sites.google.com/site/heiderawahsheh/home/web-spam-2011-datasets/arabic-web-spam-2011-dataset.

[30] Wahsheh HA, Al-Kabi MN. Detecting Arabic Web Spam. In: The 5th International Conference on Information Technology, ICIT’11; 2011.

[31] Alarifi A, Alsaleh M, Al-Salman A. Security analysis of top visited Arabic Web sites. In: Proceedings of the 15th International Conference on Advanced Communication Technology (ICACT). IEEE; 2013. p. 173–178.

[32] Guidelines for WEBSPAM-UK2007.;. http://barcelona.research.yahoo.net/webspam/datasets/uk2007/guidelines/.

[33] Svore KM, Wu Q, Burges CJ, Raman A. Improving web spam classification using rank-time features. In: Proceedings of the 3rd international workshop on Adversarial information retrieval on the web. ACM; 2007. p. 9–16.

[34] Erdélyi M, Garzó A, Benczúr AA. Web spam classification: a few features worth more. In: Proceedings of the 2011 Joint WICOW/AIRWeb Workshop on Web Quality; 2011. p. 27–34.

[35] Fetterly D, Manasse M, Najork M. Detecting phrase-level duplication on the world wide web. In: Proceedings of the 28th annual international ACM SIGIR conference on Research and development in information retrieval. ACM; 2005. p. 170–177.

[36] Piskorski J, Sydow M, Weiss D. Exploring linguistic features for web spam detection: a preliminary study. In: Proceedings of the 4th international workshop on Adversarial information retrieval on the web. ACM; 2008. p. 25–28.

[37] Becchetti L, Castillo C, Donato D, Leonardi S, Baeza-Yates RA. Link-Based Characterization and Detection of Web Spam. In: AIRWeb; 2006. p. 1–8.

[38] Spirin N, Han J. Survey on web spam detection: principles and algorithms. SIGKDD Explor Newsl. 2012;13(2):50–64. 10.1145/2207243.2207252

[39] Weka W. Weka 3: data mining software in Java. University of Waikato, Hamilton, New Zealand (www.cs.waikato.ac.nz/ml/weka). 2011;.

[40] Bouckaert RR, Frank E, Hall M, Kirkby R, Reutemann P, Seewald A, et al WEKA Manual for Version 3-7-8. Hamilton, New Zealand 2013;.

[41] Heymann P, Koutrika G, Garcia-Molina H. Fighting spam on social web sites: A survey of approaches and future challenges. IEEE Internet Computing. 2007;11(6):36–45. 10.1109/MIC.2007.125

[42] Spirin N, Han J. Survey on web spam detection: principles and algorithms. ACM SIGKDD Explorations Newsletter. 2012;13(2):50–64. 10.1145/2207243.2207252

[43] Khan WZ, Khan MK, Muhaya FTB, Aalsalem MY, Chao HC. A Comprehensive Study of Email Spam Botnet Detection. IEEE Communications Surveys & Tutorials. 2015;17(4):2271–2295. 10.1109/COMST.2015.2459015

[44] Alarifi A, Alsaleh M, Al-Salman A. Twitter turing test: Identifying social machines. Information Sciences. 2016;372:332—346. 10.1016/j.ins.2016.08.036

[45] Alsaleh M, Alarifi A, Al-Salman AM, Alfayez M, Almuhaysin A. Tsd: Detecting sybil accounts in twitter. In: Machine Learning and Applications (ICMLA), 2014 13th International Conference on. IEEE; 2014. p. 463–469.

[46] Hyun Y, Kim N. Detecting blog spam hashtags using topic modeling. In: Proceedings of the 18th Annual International Conference on Electronic Commerce: e-Commerce in Smart connected World. ACM; 2016. p. 43.

[47] Almaatouq A, Alabdulkareem A, Nouh M, Shmueli E, Alsaleh M, Singh VK, et al. Twitter: who gets caught? observed trends in social micro-blogging spam. In: Proceedings of the 2014 ACM conference on Web science. ACM; 2014. p. 33–41.

[48] Almaatouq A, Shmueli E, Nouh M, Alabdulkareem A, Singh VK, Alsaleh M, et al If it looks like a spammer and behaves like a spammer, it must be a spammer: analysis and detection of microblogging spam accounts. International Journal of Information Security. 2016; p. 1–17. 10.1007/s10207-016-0321-5

[49] Krishnan V, Raj R. Web Spam Detection with Anti-Trust Rank. In: AIRWeb. vol. 6; 2006. p. 37–40.

[50] Wu B, Goel V, Davison BD. Topical trustrank: Using topicality to combat web spam. In: Proceedings of the 15th international conference on World Wide Web. ACM; 2006. p. 63–72.

[51] Fetterly D, Manasse M, Najork M. Spam, damn spam, and statistics: Using statistical analysis to locate spam web pages. In: Proceedings of the 7th International Workshop on the Web and Databases: colocated with ACM SIGMOD/PODS 2004. ACM; 2004. p. 1–6.

[52] Wu B, Goel V, Davison BD. Propagating Trust and Distrust to Demote Web Spam. MTW. 2006;190.

[53] Castillo C, Donato D, Gionis A, Murdock V, Silvestri F. Know your neighbors: Web spam detection using the web topology. In: Proceedings of the 30th annual international ACM SIGIR conference on Research and development in information retrieval. ACM; 2007. p. 423–430.

[54] Abernethy J, Chapelle O, Castillo C. Web spam identification through content and hyperlinks. In: Proceedings of the 4th international workshop on Adversarial information retrieval on the web. ACM; 2008. p. 41–44.

[55] Becchetti L, Castillo C, Donato D, Baeza-Yates R, Leonardi S. Link analysis for web spam detection. ACM Transactions on the Web (TWEB). 2008;2(1):2 10.1145/1326561.1326563

[56] Urvoy T, Chauveau E, Filoche P, Lavergne T. Tracking web spam with html style similarities. ACM Transactions on the Web (TWEB). 2008;2(1):3 10.1145/1326561.1326564

[57] Gan Q, Suel T. Improving web spam classifiers using link structure. In: Proceedings of the 3rd international workshop on Adversarial information retrieval on the web. ACM; 2007. p. 17–20.

[58] Webb S, Caverlee J, Pu C. Introducing the Webb Spam Corpus: Using Email Spam to Identify Web Spam Automatically. In: CEAS; 2006.

[59] Lee JH, Yeh WC, Chuang MC. Web page classification based on a simplified swarm optimization. Applied Mathematics and Computation. 2015;270:13–24. 10.1016/j.amc.2015.07.120

[60] Martinez-Romo J, Araujo L. Web spam identification through language model analysis. In: Proceedings of the 5th international workshop on adversarial information retrieval on the web. ACM; 2009. p. 21–28.

